# Activity limitations predict health care expenditures in the general population in Belgium

**DOI:** 10.1186/s12889-015-1607-7

**Published:** 2015-03-19

**Authors:** Johan Van der Heyden, Herman Van Oyen, Nicolas Berger, Dirk De Bacquer, Koen Van Herck

**Affiliations:** Department of Public Health and Surveillance, Scientific Institute of Public Health, 14, Juliette Wytsmanstraat, 1050 Brussels, Belgium; Department of Public Health, Ghent University, Ghent, Belgium; Faculty of Public Health & Policy, London School of Hygiene & Tropical Medicine, London, UK

**Keywords:** Activity limitations, Disability, Health care expenditures, Chronic conditions

## Abstract

**Background:**

Disability and chronic conditions both have an impact on health expenditures and although they are conceptually related, they present different dimensions of ill-health. Recent concepts of disability combine a biological understanding of impairment with the social dimension of activity limitation and resulted in the development of the Global Activity Limitation Indicator (GALI). This paper reports on the predictive value of the GALI on health care expenditures in relation to the presence of chronic conditions.

**Methods:**

Data from the Belgian Health Interview Survey 2008 were linked with data from the compulsory national health insurance (n = 7,286). The effect of activity limitation on health care expenditures was assessed via cost ratios from multivariate linear regression models. To study the factors contributing to the difference in health expenditure between persons with and without activity limitations, the Blinder-Oaxaca decomposition method was used.

**Results:**

Activity limitations are a strong determinant of health care expenditures. People with severe activity limitations (5.1%) accounted for 16.9% of the total health expenditure, whereas those without activity limitations (79.0%), were responsible for 51.5% of the total health expenditure. These observed differences in health care expenditures can to some extent be explained by chronic conditions, but activity limitations also contribute substantially to higher health care expenditures in the absence of chronic conditions (cost ratio 2.46; 95% CI 1.74-3.48 for moderate and 4.45; 95% CI 2.47-8.02 for severe activity limitations). The association between activity limitation and health care expenditures is stronger for reimbursed health care costs than for out-of-pocket payments.

**Conclusion:**

In the absence of chronic conditions, activity limitations appear to be an important determinant of health care expenditures. To make projections on health care expenditures, routine data on activity limitations are essential and complementary to data on chronic conditions.

**Electronic supplementary material:**

The online version of this article (doi:10.1186/s12889-015-1607-7) contains supplementary material, which is available to authorized users.

## Background

Health care expenditures represent an increasing part of the GDP in all OECD countries. In Belgium the proportion of the GDP devoted to health care rose from 8.1% to 10.5% between 2000 and 2011 [1]. There is no doubt that - apart from the development of new technologies and drugs - population ageing and the associated higher burden of ill-health have contributed to this trend.

When assessing the burden of ill-health at the population level, many studies highlight the role of disability. Several frameworks exist to conceptualise disability [2,3]. Recent disability measures target functioning ability rather than impairment. Examples of measures include the Washington Group General Disability Measure [4], activities of daily living (ADLs) [5] and instrumental activities of daily living (IADLs) [6]. These standard instruments tap basic and intermediate levels of functioning, using five or more separate questions to cover the separate domains. However, the way in which functional limitations lead to activity limitations (i.e. have an impact on participation to society) also depends on external factors. This is clearly reflected in the definition of the International Classification of Functioning, Disability, and Health (ICF), in which disability is defined as a difficulty in functioning at the body, person, or societal levels, in one or more life domains, as experienced by an individual with a health condition in interaction with contextual factors [7]. This definition combines a biological understanding of impairment with the social dimension of activity restrictions [8].

The need for a global disability indicator that would capture higher-order level of functioning [9] resulted in the development of the Global Activity Limitation Indicator (GALI). The GALI identifies subjects with longstanding (at least 6 months) limitations due to a health problem by severity level via a single question [10]. The indicator refers to general restrictions in activity without specifying the type of activity (work, household chores, leisure, personal care etc.). Activity limitations due to non-health related problems are not included. The severity level relates to the activity limitations, not to the seriousness of the health problem. The GALI has been designed as a comprehensive health measure, underlying the EU indicator Healthy Life Years [11] and is used particularly for health expectancy comparisons across Europe, especially the monitoring of the progress towards the global target of two additional healthy life years (HLY) at birth by 2020 in the EU on average [12]. It is included in the European Survey on Income and Living Conditions (EU-SILC), the European Health Interview Survey (EHIS) and the Survey of Health, Ageing and Retirement in Europe (SHARE) and is therefore one of the indicators for which sustainable and comparable information is sought across all EU countries. The GALI has been validated in Belgium and other EU countries [13-18]. Both in the general population and in older people, activity limitations measured via the GALI appear to be an important predictor of short term mortality [19].

Disability is often related with the presence of chronic conditions, which include essentially physical and mental diseases, and also risk factors such as hypertension and hyperlipidaemia are chronic conditions [20]. Although the terms “chronic conditions” and “disability” are sometimes mixed up, current views prefer to consider them as related, but separate concepts [21]. People with chronic conditions may experience some form of disability, but not every person with a disability experiences a chronic health problem. Therefore in public health research, a distinction should be made between a) individuals with disabilities without any chronic condition b) individuals with disabilities who also have unrelated chronic conditions, and c) individuals with disabilities who developed a disability as a results of chronic conditions [22]. Disentangling the impact of disability and chronic conditions on health care expenditures will increase our knowledge on the need factors that lead to health care expenditures and provide evidence to health policy makers on the usefulness of routine data collection on both chronic diseases and disability for planning purposes.

To date, no studies have looked at the relation between disability and health care expenditures by making use of the GALI. Moreover, although some studies investigated the link between disability and health care expenditures, most of them were done in the US, focused on either health insurance expenses or out-of-pocket expenses and did not take into account at the same time the presence of chronic diseases and disability [23-27]. The Belgian health system is quite different from the one in the US. It is characterised by therapeutic freedom for physicians, freedom of choice for patients, a compulsory health insurance, and remuneration based on both fee-for-service payments and direct payments via sickness funds [28]. This study investigated the relation between activity limitations on health care expenditures in Belgium in relation to chronic conditions. This was assessed for the total health expenses, and for reimbursed and out-of-pocket expenses separately. Furthermore it was assessed to which extent differences in health care expenditures by activity limitation can be explained or differ by socio-demographic characteristics and prevalence of chronic conditions.

## Methods

### Data

**Figure 1:**
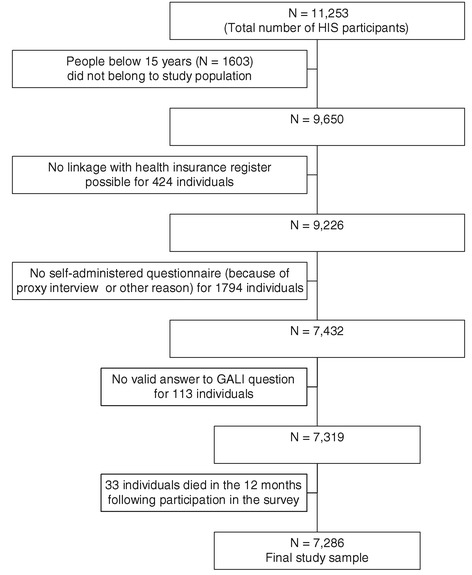
**Flow diagram of number of respondents included in the study.**

Data used in the study were from the Belgian National Health Interview Survey (HIS) 2008, which was conducted between May 2008 and July 2009 among a representative sample of Belgian residents (N = 11,253). A detailed description of the design and sampling methods can be found elsewhere [29]. The HIS data were linked to the health insurance register to obtain health care expenditures of all participants. The survey was carried out by Statistics Belgium and exempted by law from requiring ethics approval. For the use of the survey data and the linkage to the health insurance data, authorization was obtained from the Belgian Commission for the Protection of Privacy. Health insurance is compulsory in Belgium and covers more than 99% of the population. Individual linkage between the HIS and health insurance data was performed using a unique identifier. Analyses had to be restricted to 7,286 respondents aged 15 years and older for whom linkage was possible with the health insurance register, who had answered the GALI-question in the self-administered questionnaire (which was not the case for respondents interviewed by proxy) and for whom health expenditure data were available for a period of 12 months following participation to the survey (Figure 1).

Health care expenditures were obtained for the years 2008, 2009 and 2010. A distinction was made between health care expenditures for (1) ambulatory care (excluding the cost of pharmaceuticals), (2) hospital care and (3) reimbursed medicines obtained in pharmacies. For a small group of expenses, this information was not available. The database included information on health expenses covered by the health insurance, out-of-pocket expenses, and supplemental payments. Supplemental payments are additional health expenses for patients who opt for extra services, e.g. a hospital bed in a private room. In Belgium, hospitals also get a fixed amount per admitted patient from the health insurance. Information on this fixed part was not available in the database.

Activity limitations were measured with the GALI. The initial version of the question was used: “For the past 6 months or more have you been limited in activities people usually do because of a health problem? (Yes, strongly limited/Yes, limited/No, not limited)”. Information was available on the one-year prevalence of 30 specific chronic conditions and health problems. Chronic conditions were included in the multivariate model if there appeared to be an independent association with health expenditure after adjustment for age, gender and the other chronic conditions.

A composite indicator on chronic conditions was created distinguishing persons with none of the identified chronic conditions, one condition and two or more conditions. Further information used in the analyses included age, gender, educational attainment, income level, household type, nationality and degree of urbanization. Educational attainment was defined at the household level and based on the highest diploma obtained by the reference person or the partner, if any. Four categories were considered: no diploma or only primary education, lower secondary, higher secondary and tertiary education. For income level, the quintiles of the equivalent household income were calculated [30]. Five household types where distinguished: one person households, monoparental households, couples with children, couples without children and other types of households. Nationality was regrouped into Belgian, non-Belgian from an EU country and non-Belgian from outside the EU. The degree of urbanisation was based on morphological and functional characteristics of municipalities, derived from census data, which resulted into an indicator with three categories: urban, semi-urban and rural municipalities [31,32].

### Analyses

Descriptive results on overall health expenses, and health expenses by type and reimbursement modalities, were presented in function of the prevalence of activity limitations. Multivariate linear regression models were used to explore the association of health expenses with activity limitations in relation to chronic conditions and potential socio-demographic determinants. To account for the skewedness of the health care costs, the natural logarithm was used as dependent variable. One euro was added to all costs to enable a logarithmic transformation for people who had not incurred any health costs. Variability in health care expenditures was expressed as cost ratios (CR) of the logarithm of the expenses compared to a reference category [33,34].

To study the factors contributing to the difference in health expenditure between persons with and without activity limitations, the Blinder-Oaxaca decomposition method was used [35-37]. Although multivariate regression models are suitable to address differences in the importance of individual factors, the Blinder-Oaxaca technique demonstrates the relative importance of each predictor. The decomposition illustrates the fraction of the gap in health care expenditures that is attributable to group differences in the magnitude of the determinants (the explained or prevalence component) and to group differences in the effects of these determinants (the unexplained or impact component). The Blinder-Oaxaca decomposition method is particularly useful to study differences in health care expenditures between two groups [38,39], but it has also been used in studies in which the contribution of both the prevalence and the impact of determinants to explain differences between groups was investigated for other health outcomes [40,41]. Further information on the Blinder-Oaxaca decomposition is presented in an Additional file 1.

The sampling design of the Belgian HIS 2008 included stratification, clustering and over representation of the population aged 75 years and older and residents living in the Brussels-Capital Region. Analyses were done with Stata 13 [42] taking into account the design settings of the survey.

## Results

Within the study population aged 15 years and older, 5,461 individuals (79.0%) had no activity limitations, 1,364 (14.9%) reported moderate activity limitations and 461 (5.1%) severe activity limitations. Table 1 presents socio-demographic characteristics and chronic disease status of the study participants for the total sample and by level of activity limitations. It shows that chronic diseases and activity limitations are strongly related. Yet, among people with severe activity limitations, 12.8% had no chronic condition.

**Table 1:** **Socio-demographic characteristics and chronic disease status of study participants by level of activity limitation (AL)**

	**Total**		**No AL**		**Moderate AL**		**Severe AL**	
	**%** ^**§**^	**N**	**%** ^**§**^	**N**	**%** ^**§**^	**N**	**%** ^**§**^	**N**
**Gender**								
Men	47.9	3,353	49.8	2,638	41.4	552	38.2	163
Women	52.1	3,933	50.2	2,823	58.6	812	61.8	298
**Age (in years)**								
15-24	13.1	869	15.9	828	2.9	31	2.2	10
25-44	33.5	2,238	37.9	1,973	17.3	205	14.7	60
45-64	33.7	2,260	31.9	1,674	41.1	453	38.2	133
65+	19.8	1,919	14.3	986	38.7	675	44.9	258
**Education**								
No diploma/only primary	10.4	904	7.8	489	19.2	282	25.0	133
Lower secondary	15.4	1,161	13.5	756	22.6	285	23.8	120
Higher secondary	33.3	2,260	33.9	1,744	31.5	402	29.8	114
Tertiary	40.8	2,797	44.9	2,360	26.8	357	21.5	80
**Income**								
Quintile 1	17.5	1,192	16.3	820	21.9	277	22.0	95
Quintile 2	17.8	1,231	15.3	786	23.4	307	36.5	138
Quintile 3	19.3	1,144	18.5	826	22.0	232	22.4	86
Quintile 4	21.8	1,223	23.5	992	17.4	182	10.8	49
Quintile 5	23.7	1,427	26.4	1,203	15.3	188	8.4	36
**Nationality**								
Belgian	92.5	6,513	91.9	4,827	94.2	1,255	95.5	431
Non-Belgian EU	5.3	542	5.6	440	4.3	79	3.4	23
Non-Belgian non-EU	2.3	211	2.5	178	1.5	27	1.1	6
**Household type**								
Single	18.0	1,922	15.2	1,220	26.6	494	33.8	208
One parent with child(ren)	7.6	580	7.9	463	7.0	84	5.7	33
Couple with child(ren)	26.7	1,859	24.1	1,274	36.7	445	37.4	140
Couple without child(ren)	37.1	2,248	42.0	1,985	19.4	211	15.2	52
Other or unknown	10.6	677	10.9	519	10.3	130	7.9	28
**Degree of urbanisation**								
Urban	45.9	3,946	44.7	2,911	49.3	777	53.5	258
Semi-urban	23.1	1,292	23.3	966	23.1	244	20.1	82
Rural	31.0	2,048	32.0	1,584	27.5	343	26.4	121
**Chronic disease status**								
No chronic condition^#^	56.5	3,695	66.4	3,380	21.5	256	12.8	59
1 chronic condition^#^	22.7	1,632	21.4	1,167	28.5	362	24.6	103
≥2 chronic conditions^#^	20.8	1,710	12.3	728	50.0	697	62.6	285

^§^Weighted percentage.

^#^The following chronic conditions are considered: asthma, chronic bronchitis, myocardial infarction, coronary heart disease, stroke, hypertension, osteoarthritis, neck disorder, depression, peptic ulcer, problem large bowel, diabetes, thyroid problems, kidney problems except for kidney stones, cancer.

People with severe activity limitations accounted for 16.9% of the total health expenditure, whereas those without activity limitations were responsible for 51.5% of the total health expenditure. On average 57.0% of the expenses were ambulatory costs, 21.8% hospital costs (excluding fixed costs, as explained in the methods section), 18.2% costs for reimbursed medicines obtained in pharmacies and 3.0% not specified. The large majority of expenses (84.0%) were covered by the health insurance, 10.9% were out-of-pocket payments and 5.1% supplements.

Table 2 shows descriptive results of the mean health care expenditures in the 12 months following the participation of the survey. Overall average yearly health care expenditures increased from 1,220 euro per year among people with no limitation, over 3,803 euro among people with moderate limitations to 7,358 euro among people with limitations.

**Table 2:** **Mean annual health expenses (in euro) in function of activity limitation**

	**Not limited (n = 5,461)**		**Moderately limited (n = 1,364)**		**Severely limited (n = 461)**		**All (n = 7,286)**	
	**Mean**	**(95% CI)**	**Mean**	**(95% CI)**	**Mean**	**(95% CI)**	**Mean**	**(95% CI)**
**By type of health expenses**								
Ambulatory care (no medicines)	713	(662-764)	2,143	(1,869-2,417)	3,989	(3,342-4,636)	1,108	(1,034-1,181)
Hospital care	257	(189-326)	784	(627-941)	1,748	(1,221-2,275)	417	(351-484)
Reimbursed medicines obtained in pharmacies	220	(201-239)	770	(668-872)	1,370	(1,042-1,699)	366	(336-396)
Not specified	32	(27- 37)	116	(96-137)	280	(192-369)	58	(51-65)
**By payment modality**								
Health insurance	998	(898-1,098)	3,283	(2,941-3,624)	6,614	(5,658-7,570)	1,648	(1,530-1,766)
Out-of-pocket	158	(149-167)	374	(346-402)	567	(476-658)	213	(203-224)
Supplement	64	(55-74)	147	(108-185)	178	(103-254)	83	(73-94)
**All health expenses**	1,220	(1,110-1,329)	3,803	(3,435-4,170)	7,358	(6,309-8,408)	1,944	(1,815-2,073)

Although the mean values are highly influenced by the individuals with high expenditures, the median values also differ substantially by level of activity limitations: for people with no limitation the median was 506 euro; for people with moderate and severe limitations median expenditures were 1,965 euro and 3,768 euro, respectively. The contribution of reimbursed health care expenditures increased with the severity of limitations: from 81.8% if no limitations are reported to 86.3% in case of moderate and 89.9% in case of severe limitations. The differences are significant (p < 0.001) after adjustment for age and gender.

Fifteen out of 30 chronic conditions were significantly associated with higher health care expenditures after adjustment for age, gender and the other chronic conditions (Table 3).

**Table 3:** **Prevalence of chronic conditions considered in the study and cost ratio (CR) of total health expenses for persons with the disease compared to those without**

		**Prevalence (weighted %) (95% CI)**	**CR** ^**§**^ **(95% CI)**
**Respiratory problems**			
	Asthma	4.1 (3.6-4.8)	1.44* (1.15-1.81)
	Chronic bronchitis	3.9 (3.4-4.5)	1.33* (1.06-1.68)
**Cardiovascular problems**			
	Myocardial infarction	0.7 (0.5-0.9)	1.90* (1.28-2.84)
	Coronary heart disease	2.1 (1.7-2.6)	1.92* (1.33-2.78)
	Stroke	1.0 (0.7-1.3)	1.36* (1.01-1.82)
	Hypertension	15.9 (14.7-17.1)	1.50* (1.30-1.73)
**Musculoskeletal problems**			
	Rheumatoid arthritis	7.2 (6.5-8.0)	1.12 (0.92-1.37)
	Osteoarthritis	16.0 (14.8-17.2)	1.19* (1.00-1.40)
	Low back disorder	21.1 (19.7-22.4)	1.08 (0.91-1.27)
	Neck disorder	11.9 (10.9-12.9)	1.22* (1.03-1.44)
	Osteoporosis	4.6 (4.1-5.3)	0.83 (0.64-1.07)
**Neurological problems**			
	Headache/migraine	10.0 (9.1-11.9)	1.15 (0.95-1.39)
	Parkinson’s disease	0.4 (0.2-0.6)	2.00 (0.60-6.71)
	Epilepsy	0.6 (0.4-1.0)	1.69 (0.88-3.22)
**Psychological problems**			
	Chronic anxiety	5.2 (4.6-6.0)	1.23 (0.99-1.53)
	Depression	6.0 (5.3-6.8)	1.78* (1.44-2.20)
**Problems related to digestive system**			
	Peptic ulcer	3.7 (3.1-4.4)	1.62* (1.22-2.15)
	Liver problems	0.5 (0.4-0.7)	1.61 (0.76-3.39)
	Problems of the large bowel	3.2 (2.7-3.8)	1.43* (1.10-1.88)
	Gall-bladder problems	0.7 (0.5-0.9)	0.96 (0.58-1.62)
**Problems related to endocrine system**			
	Diabetes	3.8 (3.3-4.4)	2.31* (1.91-2.79)
	Thyroid problems	4.5 (4.0-5.2)	1.30* (1.04-1.62)
**Urinary problems**			
	Urinary incontinence	3.3 (2.8-3.9)	1.24 (0.93-1.65)
	Chronic cystitis	1.3 (1.0-1.6)	1.13 (0.83-1.54)
	Kidney stones	0.7 (0.5-1.0)	0.88 (0.37-2.10)
	Other kidney problems	0.7 (0.5-0.9)	2.73* (1.22-6.09)
**Eye problems**			
	Glaucoma	1.7 (1.4-2.1)	1.17 (0.86-1.58)
	Cataract	2.8 (2.4-3.3)	1.00 (0.78-1.28)
**Other problems or diseases**			
	Cancer (all types)	2.1 (1.7-2.6)	3.77* (2.56-5.57)
	Allergy	14.0 (12.8-15.2)	1.04 (0.88-1.22)
	Chronic or severe skin disease	3.0 (2.4-3.7)	1.26 (0.94-1.69)

^§^adjusted for age, gender and the other diseases, *significant (p < 0.05).

The association was highest for cancer (CR 3.77), kidney problems other than kidney stones (CR 2.73) and diabetes (CR 2.31), and was also significant, in descending order of the magnitude of the association, for coronary heart disease (CR 1.92), myocardial infarction (CR 1.90), depression (CR 1.78), peptic ulcer (CR 1.62), hypertension (CR 1.50), asthma (CR 1.44), problems of the large bowel (CR 1.43), stroke (CR 1.36), chronic bronchitis (CR 1.33), thyroid problems (CR 1.30), neck disorder (CR 1.22) and osteoarthritis (CR 1.19).

Table 4 presents differences in health expenditure by level of activity limitation and by number of chronic conditions after adjustment for age, gender, education, income, nationality, household type and degree of urbanisation. Both activity limitations and chronic conditions contribute independently to health expenditure. The adjusted CR was 1.88 and 3.21 in people with respectively moderate and severe limitations. People with one chronic condition (among the 15 that were selected) had an adjusted cost ratio of 1.82 compared to those without any of those diseases; for those with more than one chronic conditions the CR was 2.84. Chronic conditions and activity limitations contributed in a cumulative way to increase health care expenditures. In the absence of an activity limitation, the adjusted CR for those with one chronic condition was 1.94 and for those with two or more chronic conditions 3.01. Among persons with no chronic condition, the adjusted CR was 2.46 if they had a moderate activity limitation and 4.45 in case of a severe activity limitation. However, a severe activity limitation in combination with more than one chronic condition yielded an adjusted CR of 9.60. The interaction between activity limitation and chronic condition was not significant (results not shown), which indicates that the association between activity limitation and health care expenditure did not depend on the presence of a chronic condition.

**Table 4:** **Cost ratios (CR) of health expenses by activity limitations and chronic conditions** ^**§**^ **, adjusted for age, gender, education, income, nationality, household type and degree of urbanisation, in function of payment modalities**

		**Total health expenditure**		**Covered by health insurance**		**Out-of-pocket**		**Supplement**	
		**CR**	**95% CI**	**CR**	**95% CI**	**CR**	**95% CI**	**CR**	**95% CI**
**Activity limitations adjusted for chronic conditions**									
No limitation		1.00		1.00		1.00		1.00	
Moderate activity limitation		1.88	(1.59-2.22)	1.91	(1.62-2.56)	1.54	(1.35-1.76)	1.64	(1.33-2.03)
Severe activity limitation		3.21	(2.57-4.02)	3.41	(2.72-4.27)	2.03	(1.67-2.48)	2.14	(1.52-3.01)
**Chronic condition adjusted for activity limitations**									
No chronic condition^#^		1.00		1.00		1.00		1.00	
1 chronic condition^#^		1.82	(1.57-2.11)	1.81	(1.56-2.09)	1.76	(1.56-1.99)	1.26	(1.05-1.52)
≥2 chronic conditions^#^		2.84	(2.41-3.35)	2.87	(2.43-3.38)	2.51	(2.19-3.87)	1.51	(1.23-1.89)
**Activity limitations and chronic conditions combined**									
No activity limitation - no chronic condition^#^		1.00		1.00		1.00		1.00	
No activity limitation – 1 chronic condition^#^		1.94	(1.66-2.27)	1.93	(1.65-2.26)	1.86	(1.63-2.11)	1.27	(1.04-1.55)
No activity limitation – ≥2 chronic conditions^#^		3.01	(2.48-3.65)	3.04	(2.51-3.69)	2.59	(2.20-3.04)	1.33	(1.04-1.71)
Moderate activity limitation - no chronic condition^#^		2.46	(1.74-3.48)	2.50	(1.77-3.54)	1.82	(1.36-2.44)	1.46	(1.02-2.09)
Moderate activity limitation – 1 chronic condition^#^		3.16	(2.38-4.20)	3.21	(2.43-4.25)	2.59	(2.06-3.25)	2.06	(1.29-3.04)
Moderate activity limitation - ≥2 chronic conditions^#^		5.20	(4.23-6.39)	5.33	(4.34-6.55)	3.82	(3.26-4.48)	2.55	(1.87-3.48)
Severe activity limitation - no chronic condition^#^		4.45	(2.47-8.02)	4.70	(2.57-8.58)	2.82	(1.66-4.79)	1.21	(0.60-2.43)
Severe activity limitation – 1 chronic condition^#^		4.80	(2.99-7.70)	5.14	(3.20-8.25)	2.92	(1.92-4.45)	1.87	(1.00-3.51)
Severe activity limitation - ≥ 2 chronic conditions^#^		9.60	(7.41-12.44)	10.22	(7.87-13.27)	5.31	(4.24-6.64)	4.11	(2.68-6.29)
**Activity limitations – stratified analysis**									
No chronic condition^#^	No activity limitation	1.00		1.00		1.00		1.00	
	Moderate activity limitation	2.47	(1.73-3.52)	2.51	(1.77-3.58)	1.82	(1.36-2.45)	1.45	(1.01-2.07)
	Severe activity limitation	4.21	(2.43-7.32)	4.46	(2.54-7.85)	2.66	(1.62-4.36)	1.16	(0.57-2.35)
1 chronic condition^#^	No activity limitation	1.00		1.00		1.00		1.00	
	Moderate activity limitation	1.51	(1.14-2.00)	1.54	(1.17-2.04)	1.32	(1.05-1.66)	1.59	(1.07-2.34)
	Severe activity limitation	2.42	(1.53-3.81)	2.60	(1.65-4.11)	1.57	(1.04-2.35)	1.50	(0.82-2.73)
≥ 2 chronic conditions^#^	No activity limitation	1.00		1.00		1.00		1.00	
	Moderate activity limitation	1.72	(1.39-2.14)	1.75	(1.40-2.17)	1.49	(1.26-1.77)	1.90	(1.35-2.70)
	Severe activity limitation	3.21	(2.49-4.14)	3.37	(2.60-4.36)	2.12	(1.70-2.65)	2.98	(1.91-4.63)

^§^Defined as: having suffered in the past 12 months from at least one of the following health problems: asthma, chronic bronchitis, myocardial infarction, coronary heart disease, stroke, hypertension, osteoarthritis, neck disorder, depression, peptic ulcer, problem large bowel, diabetes, thyroid problems, kidney problems except for kidney stones, cancer.

^#^Limited to list of chronic conditions specified above.

This was confirmed in a stratified analysis, separating respondents with no, one and two or more chronic conditions. In the group with no chronic conditions, the adjusted CR of health expenses was 2.47 for people with moderate activity limitations and 4.21 for people with severe activity limitations. Also in the two groups with chronic diseases there was a clear association between health expenses and activity limitations.

It is striking that differences in cost ratios are far more pronounced for payments covered by the health insurance than for out-of-pocket expenses and supplements. The adjusted CR for people with a severe activity limitation and more than one chronic condition compared to those with none of both is much higher (10.22) for payments covered by the health insurance, than for out-of-pocket payments (5.31) and supplements (4.11).

The decomposition analysis (Table 5) shows that 47.9% (0.768/1.605; p < .001) of the difference in health care expenditures between people with and without activity limitations can be explained by variation in the distribution of socio-demographic characteristics and prevalence of chronic conditions, meaning that almost half of the gap in health care expenditures would be mitigated if people with activity limitations would have the same characteristics as people without activity limitations. The difference in impact of those characteristics, which corresponds to the unexplained gap (meaning the change in health care expenditures among people with activity limitations when applying coefficients of people without activity limitations to those with activity limitations) accounts for 52.1% (0.837/1.605; p < 0.001) of the gap in health care expenditures. Age, gender and education influence significantly the gap via both the prevalence and the impact. Differences in the prevalence of chronic conditions between people with and without activity limitations explain 22.2% (0.356/1.605; p < 0.001) of the gap in health care expenditures, but the impact of chronic conditions on people with activity limitations does not yield significantly different health care expenditures than in people without activity limitations (p = 0.260).

**Table 5:** **Decomposition of the gap between people with activity limitations (AL) and no activity limitations in terms of the natural logarithm of the health care expenditure and the contribution of each variable in creating this gap in the explained and unexplained components**

**Log (health care expenditure)**	**Total health expenditure**		**Covered by health insurance**		**Out-of-pocket**		**Supplement**	
	**Coeff.**	**p-value**	**Coeff.**	**p-value**	**Coeff.**	**p-value**	**Coeff.**	**p-value**
**Prediction in people with AL**	7.499	<0.001	7.319	<0.001	5.379	<0.001	2.245	<0.001
**Prediction in people without AL**	5.894	<0.001	5.662	<0.001	4.201	<0.001	1.395	<0.001
**Gap**	1.605	<0.001	1.657	<0.001	1.178	<0.001	0.850	<0.001
**Explained (prevalence)**								
Age	0.324	<0.001	0.333	<0.001	0.279	<0.001	0.143	<0.001
Gender	0.056	<0.001	0.053	<0.001	0.053	<0.001	0.037	<0.001
Education	0.049	0.030	0.054	0.015	0.026	0.175	−0.038	0.152
Income	−0.010	0.564	−0.005	0.751	−0.038	0.011	−0.036	0.072
Nationality	0.018	0.099	0.016	0.110	0.015	0.042	0.004	0.783
Household type	−0.025	0.265	−0.019	0.402	−0.044	0.017	0.007	0.889
Degree of urbanisation	0.002	0.706	0.001	0.749	0.002	0.549	0.001	0.256
Chronic condition	0.356	<0.001	0.356	<0.001	0.324	<0.001	0.141	<0.001
Total	0.768	<0.001	0.791	<0.001	0.616	<0.001	0.259	<0.001
**Unexplained (impact)**								
Age	0.882	0.023	0.871	0.026	0.586	0.033	0.605	0.109
Gender	−0.635	0.003	−0.616	0.004	−0.516	0.002	0.010	0.973
Education	0.347	0.029	0.337	0.034	0.271	0.040	0.334	0.108
Income	−0.118	0.394	−0.128	0.357	0.009	0.936	−0.007	0.966
Nationality	−0.004	0.852	−0.005	0.837	0.002	0.907	0.018	0.570
Household type	−0.284	0.035	−0.283	0.035	−0.090	0.386	−0.084	0.722
Degree of urbanisation	−0.031	0.649	−0.029	0.669	−0.036	0.502	−0.032	0.304
Chronic condition	−0.151	0.260	−0.149	0.271	−0.075	0.482	0.233	0.129
Constant	0.830	0.118	0.867	0.104	0.410	0.295	−0.486	0.385
Total	0.837	<0.001	0.866	<0.001	0.562	0.000	0.591	<0.001

The decomposition analysis reveals more or less similar results for health care expenditures covered by the health insurance, out-of-pocket payments and supplements. Nevertheless, the fraction of the gap in health care expenditures between people with and without activity limitations that can be explained by differences in socio-demographic characteristics and prevalence of chronic conditions is smaller for supplements (30.5%) than for health care expenditures covered by the health insurance (47.7%) and out-of-pocket payments (52.3%).

## Discussion

This study aimed to investigate the predictive value of the GALI on health care expenditures in relation to the presence of chronic conditions. Our findings indicate that activity limitations, as measured by the GALI, are a strong determinant of health care expenditures. To some extent differences in health care expenditures by level of activity limitations can be explained by chronic conditions. However, also after controlling for chronic conditions, there is a strong and independent association between activity limitations and health care expenditures.

From a conceptual point of view the GALI is a measure of participation (about functioning at the societal level), rather than an indicator on functional limitations (limitations with basic actions such walking, climbing steps, reading, communication). It embodies the social model of disability, which has also influenced the current WHO classification (ICF) [43]. In this model the availability of facilities aiming at a better participation of people with functional limitations into society is an essential element. As current views on disability focus increasingly on this social model, it is important that measures taking into account this component are used in disability research.

The health care expenditures for which the research questions were investigated include the majority of health expenses. Fixed hospital costs, representing about a fifth of the total expenditure, were not included. Information was also available for out-of-pocket and supplemental payments. Due to the compulsory nature of the Belgian health insurance and the representativeness of the sample, extrapolation of the study results to the total Belgian population is warranted.

The descriptive results clearly demonstrate the impact of activity limitations on health care expenditure in absolute terms. Although the context, the target group and the concept of disability differ from a study conducted in the US in 2007–2008 [27], it is remarkable that the median overall health care expenditures earlier reported are close to our findings (4234 US$ for persistent disability, 1612 US$ for temporary disability and 748 US$ for no disability). Another US study [26] also reports values that are in line with the figures of our study.

Although the population with activity limitations represents only one fifth of the population, it accounts for almost half of the health care expenditures. If a causal relationship is assumed, a reduction of activity limitations in the population could lead to considerable savings.

The mechanism through which activity limitations lead to higher health care expenses cannot solely be explained in terms of differences in chronic conditions. Most studies exploring differences in health care expenditures focus either on disability, either on chronic conditions [44]. However, the conceptual framework of disability currently preferred by most experts is one in which disability is related to, but separate from chronic conditions [21]. There are several reasons for this. First, the disabling impact of different chronic conditions varies [45]. Musculoskeletal diseases contribute a lot to the burden of disability; for well treated hypertension the disabling effect is negligible. Second, disentangling disability and chronic conditions helps to highlight the social dimension of disability as an explanatory factor for differences in health care expenditures. Our results clearly indicate that chronic conditions explain to a certain extent, but definitely not completely, differences in health care expenditures by level of activity limitation. Although the independent association between activity limitations and health care expenditures, observed in this study, may have been partially due to chronic conditions which were neither in the list, nor reported, the strength of the association suggests that an important fraction of health care expenditures is generated by health related activity limitations among people with no chronic conditions. Maybe there is link with acute health problems or disabilities since birth that are not associated with chronic conditions. A better understanding of the nature and causes of those activity limitations may help health policy makers identify areas on which they should focus to improve population health and reduce health care expenditures. Third, health care expenditures are also associated with chronic conditions without or with only moderate activity limitations. Unfortunately our study does not allow concluding that the health care provided to those patients controls their chronic conditions to such an extent that they are able to participate on an equal basis in social roles and activities. Further research addressing this issue would be very interesting.

The fraction of the population with zero health care expenses was in our study minimal (only 5.7%) and the log transformation of the health care expenses corrected for the skewness of the distribution. Therefore the use of linear regression was appropriate. A similar approach has been used in other studies investigating the association between disability and health care costs [23].

In our study we used the Blinder-Oaxaca decomposition method to try to explain differences in health care expenditures. Although multivariate regression models are suitable to address differences in the importance of individual predictors, decomposing techniques demonstrate the relative importance of each predictor. The decomposition illustrates the fraction of the gap in health care expenditures that is attributable to group differences in the magnitudes of the determinants, which is the prevalence effect, and to group differences in the effects of these determinants, which is the impact effect. The Blinder-Oaxaca decomposition method is particularly useful to study differences in health care expenditures between two groups [38,39], but it has also been used in studies in which the contribution of both the prevalence and the impact of determinants to explain differences between groups was investigated for other health outcomes [40,41].

The principal finding from the decomposition analysis is that the gap in health care expenditures between people with and without activity limitations is not significantly affected by a different impact of chronic conditions. This finding confirms that chronic conditions and activity limitations are different concepts that need to be disentangled.

The association between activity limitations and health care expenditures is stronger for health care expenditures covered by the health insurance than for out-of-pocket payments. As activity limitations can be considered a need factor, this finding reflects that there is a clearer link between need and use of public health resources than between need and own contributions. It supports the hypothesis that strategies for the reimbursement of health care expenditures in Belgium, aiming at an efficient and rational use of public health funds, are quite successful.

Supplemental payments are not only related to health needs, but also to extra patient comfort. Therefore it is not surprising that a weaker association is found with activity limitations than this is the case for the other types of health care expenses. Probably this is also the reason why the fraction of the gap in health care expenditures by activity limitation that is explained by socio-demographic characteristics and prevalence of chronic conditions is smaller for supplemental payments than for the other types of health care expenses.

To our knowledge this is the first published study to investigate differences in health care expenditures by disability status in a representative sample of a European country. The Belgian health system is based on a Bismarckian model, which is essentially characterised by a premium financed social insurance system with a mixture of public and private providers. This is opposed to the Beveridge model in the UK and the Scandinavian countries, based on taxation with many public providers, and the private insurance model of the US [46]. The extent to which the type of health system interferes with the observed results is unknown. Probably the effect of the health system is minor. Studies in the US including disability measures that essentially measured functional status report results that are quite comparable with ours.

Our findings are also important from a health policy perspective. In the planning of strategies for health care cost containment, policymakers should not only consider the impact of chronic diseases on health care expenditures, but also be aware of the role of disability and its consequences. Reducing activity limitations in the population, e.g. by measures that facilitate the participation of people with functional limitations in the society, is cost effective, not only because it increases the quality of life and the productivity of people, but also because of its direct impact on the health care costs. 

Some study limitations should be reported. Although the initial study sample was representative of the total population, some groups had to be excluded: people for whom no linkage could be done (4.4%), people for whom no self-administered questionnaire was available, mostly because of a proxy interview (18.6%), people who did not answer the GALI question (1.2%) and respondents who died in the 12 months following participation in the survey (less than 0.5% of the total). Even though it is known that health care expenditures increase drastically during the last year of life [47], it is assumed that the exclusion of the latter group did not affect the results substantially, because of its small size. In contrast, the exclusion of proxy interviews, in which information on the GALI is lacking, may have had a bigger impact on the results. Respondents interviewed by proxy are probably in worse health, and therefore have higher health care expenditures, than self-respondents. Whereas this may have influenced the level of the health care expenditures, it is not sure that this had an impact on the associations that were investigated.

Some health care expenditures were not included, e.g. fixed day fees for a hospital stay. The same applies for health care expenditures that were not reported within the compulsory health insurance system, although these represent only a marginal fraction in comparison with the reported health care expenditures.

Information on chronic conditions was based on self-reports. The validity of self-reported specific chronic diseases is limited and strongly depends on the type of disease [48,49]. Furthermore chronic conditions included in the study were restricted to the list of diseases available in the Belgian HIS 2008.

Measuring disability is challenging. Although the GALI has been validated, it remains a self-reported item, with several crucial conceptual elements included in one question. There may be concerns about the accuracy in which respondents answer the question. However, the results of this manuscript add evidence for the predictive validity of the measure.

## Conclusion

Activity limitations, both moderate and severe, are a major driver for health care expenditures. This is particularly the case for reimbursed health care expenditures. Chronic conditions explain to a certain extent differences in health care expenditures by level of activity limitation. However, also in the absence of chronic conditions, activity limitations appear to be an important determinant of health care expenditures. To make projections on health care expenditures, routine data on activity limitations are essential and complementary to data on chronic conditions.

## Additional file

Additional file 1:
**The Blinder-Oaxaca decomposition method.**
