# Supplementary material for: Activity limitations predict health care expenditures in the general population in Belgium
Source: BMC Public Health. 2015 Mar 19;15:267. doi: 10.1186/s12889-015-1607-7 (PMC4409706; doi:10.1186/s12889-015-1607-7)
Supplement: Additional file 1: — The Blinder-Oaxaca decomposition method. [file 12889_2015_1607_MOESM1_ESM.pdf]

Additional material

The Blinder-Oaxaca decomposition method

The counterfactual decomposition technique described by Blinder and Oaxaca is often used to analyse wage gaps by sex or race. It ”decomposes” outcome variables into explained and unexplained variation. This “unexplained” part is often used as a measure for discrimination, but it also subsumes the effects of group differences in unobserved predictors. Most applications of the technique can be found in the labour market and discrimination literature, but the method may also be useful in other fields. In this study, it was applied to analyse differences in health care expenditures by activity limitation status.

The assumptions of the Blinder-Oaxaca decomposition are that the outcome variable y is linearly related to the covariates, x, and the error term, ε, is independent of x. It is assumed that the difference in health care expenditures, y, between people with and without activity limitation is explained by a vector of determinants, x, in a regression model. First, separate regression models are constructed for people with and without activity limitation

$$y_{NAL} = \alpha_{NAL} + \beta_{NAL}\, x_{NAL} + \varepsilon_{NAL} \quad (1)$$
$$y_{AL} = \alpha_{AL} + \beta_{AL}\, x_{AL} + \varepsilon_{AL} \quad (2)$$

To look at differentials in health care expenditures between people with and without activity limitations, a counterfactual equation is constructed by replacing the intercept and the coefficient in the first equation (1) with those from the second one (2):

$$y_{NAL}{*} = \alpha_{AL} + \beta_{AL}\, x_{NAL} + \varepsilon_{NAL}$$

The differential can then be split up between a difference due to characteristics effects (explained variation) and coefficient effects (unexplained variation):

$$\bar{y}_{AL} - \bar{y}_{NAL} = (\bar{y}_{AL} - \bar{y}_{NAL}{*}) + (\bar{y}_{NAL}{*} - \bar{y}_{NAL})$$

with the explained variation $\bar{y}_{AL} - \bar{y}_{NAL}{*} = \beta_{NAL}\,(\bar{x}_{AL} - \bar{x}_{NAL})$
and the unexplained variation $\bar{y}_{NAL}{*} - \bar{y}_{NAL} = (\alpha_{AL} - \alpha_{NAL}) + (\beta_{AL} - \beta_{NAL})\,\bar{x}_{NAL}$
which results in the following Blinder-Oaxaca decomposition equation:

$$\bar{y}_{AL} - \bar{y}_{NAL} = \beta_{AL}\,(\bar{x}_{AL} - \bar{x}_{NAL}) + [(\alpha_{AL} - \alpha_{NAL}) + (\beta_{AL} - \beta_{NAL})\,\bar{x}_{NAL}] \quad (3)$$

A graphical illustration is presented in Figure 2.

Figure 2 Health care expenditures (y) in function of a vector of determinants (x) by activity limitation. Graphical illustration of the Blinder-Oaxaca decomposition

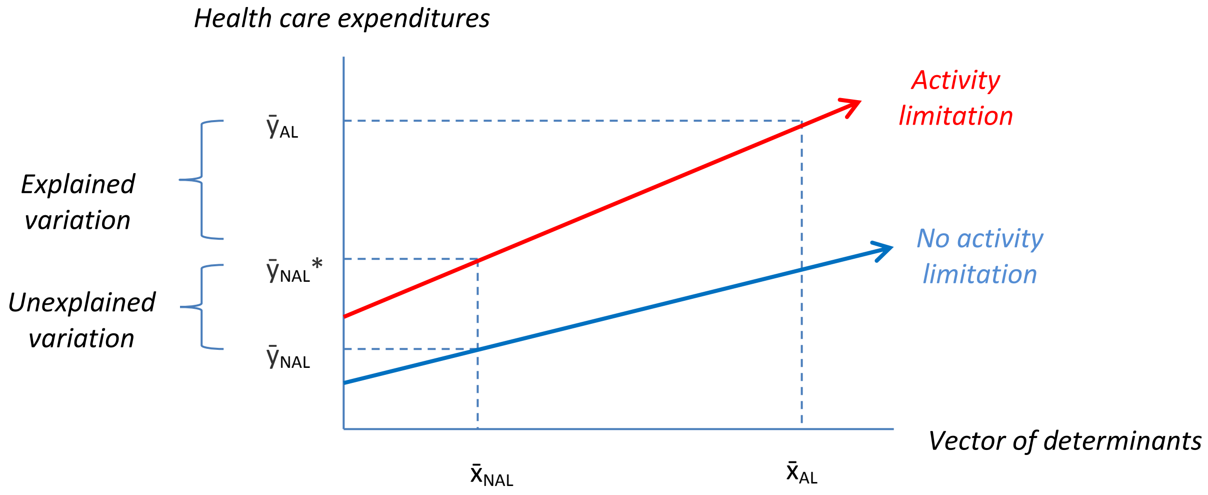

Often not only is the total decomposition of the outcome differential into an explained and un explained part of interest, but also the detailed contributions of the single predictors or sets of predictors are subject to investigation. Identifying the contributions of the individual predictors to the explained part of the differential is easy because the total component is a simple sum over the individual contributions. For example, for decomposition (3),

$$\beta_{AL} (\bar{x}_{AL} - \bar{x}_{NAL}) = \beta_{1AL} (\bar{x}_{1AL} - \bar{x}_{1NAL}) + \beta_{2AL} (\bar{x}_{2AL} - \bar{x}_{2NAL}) + \ldots$$

where $\bar{x}_1$, $\bar{x}_2$,…. are the means of the single regressors and β_1, β_2,… are the associated coefficients. The first summand reflects the contribution of the group differences in $\bar{x}_1$,the second of differences in $\bar{x}_2$, and so on. Similarly, using decomposition (3) as an example, the individual contributions to the unexplained part are the summands in

$$(\alpha_{AL} - \alpha_{NAL}) + (\beta_{AL} - \beta_{NAL}) \bar{x}_{NAL} = [(\alpha_{1AL} - \alpha_{1NAL}) + (\beta_{1AL} - \beta_{1NAL}) \bar{x}_{1NAL}] + [(\alpha_{2AL} - \alpha_{2NAL}) + (\beta_{2AL} - \beta_{2NAL}) \bar{x}_{2NAL}] + \ldots$$
